# Early Clinical Mapping of Submandibular Gland Fistula: A Case Report and Systematic Review

**DOI:** 10.1055/s-0043-1767801

**Published:** 2023-10-06

**Authors:** Vivek Dokania, Md Ibrar, Mayashankar Vishwakarma

**Affiliations:** 1 Department of Otolaryngology- Head & Neck Surgery, Asian Super Specialty Hospital, Dhanbad, Jharkhand, India; 2 Department of Radiology, Asian Super Specialty Hospital, Dhanbad, Jharkhand, India; 3 Department of Otolaryngology- Head & Neck Surgery, Khan Bahadur Bhabha Hospital, Kurla, Mumbai, Maharashtra, India

**Keywords:** salivary gland, fistula, submandibular gland

## Abstract

**Introduction** Submandibular gland fistula (SGF) is a rare subset of salivary gland fistulas. It is seldom tough to diagnose them prior to surgical exploration, and it is often clinically confused with close differentials. An early diagnosis based on pertinent clinical features and focused radiological findings can be pivotal in optimal management and help prevent recurrence and avoid unnecessary investigations/interventions.

**Objective** To review articles that discuss SGF and provide vital etiological, clinical, and imaging features of this rare entity that can aid in early clinical diagnosis.

**Data Synthesis** An extensive review involving PubMed and Google Scholar and reported in accordance with the Preferred Reporting Items for Systematic Reviews and Meta-Analyses (PRISMA) standards.

**Conclusion** Submandibular gland fistula is a rare entity. It can be confused with close differentials, including branchial fistulas, if not thoroughly examined. Discharge from fistulae along with submandibular pain/tenderness and/or swelling are important diagnostic clues. A history of trauma, nodule at the site of discharge, prior submandibular disease/calculi, or discharge aggravated with food further increases a clinical suspicion. Optimal radiological investigation looking for calculi/foreign body and delineating the fistula tract is vital to affirm a diagnosis. Gland with fistula excision is a commonly advocated treatment of choice with no reports of recurrence, although conservative management and gland preserving surgery have also reported a favorable prognosis.

## Introduction

Branchial fistulas are the most common differential for a lateral neck fistula. 1 Salivary gland fistulas are relatively less common and are mostly limited to the parotid gland. 2 A submandibular gland fistula (SGF) is extremely rare and can often be clinically confused with branchial fistula and other close differentials.

Various etiologies have been attributed to SGF, including both congenital and acquired. 3 4 5 6 7 8 9 10 11 12 13 14 15 16 17 18 The clinical manifestation of SGF varies and is influenced by the etiology of the disease, making it tough for clinicians to diagnose it clinically. Furthermore, the site of the fistula opening has been shown to have an effect on the clinical features.

Certain clinical clues and radiological findings can help achieve a confirmatory diagnosis even prior to surgical exploration or histopathological examination. This will aid clinicians in optimal surgical planning and avoid irrelevant investigations/interventions.

## Review of Literature

### Case presentation

**Fig. 1:**
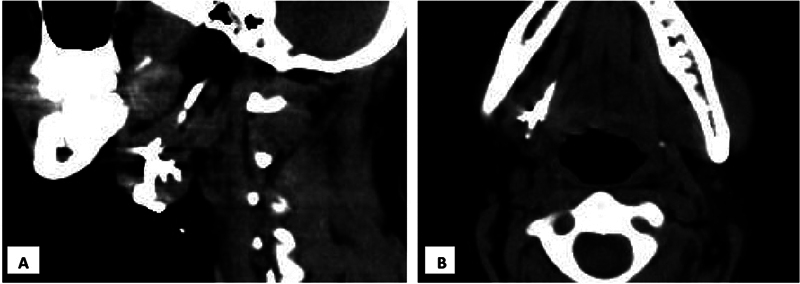
Important computed tomography fistulography findings. (A) Sagittal view: a vertical tract extending from the neck skin to the intraglandular parenchyma corresponding with sialo-cutaneous fistula, and a horizontal tract converging from the intraglandular region that represents the Wharton duct. (B) Axial view: Horizontal tract extending from the submandibular gland till the floor of the mouth, consistent with Wharton duct.

**Fig. 2:**
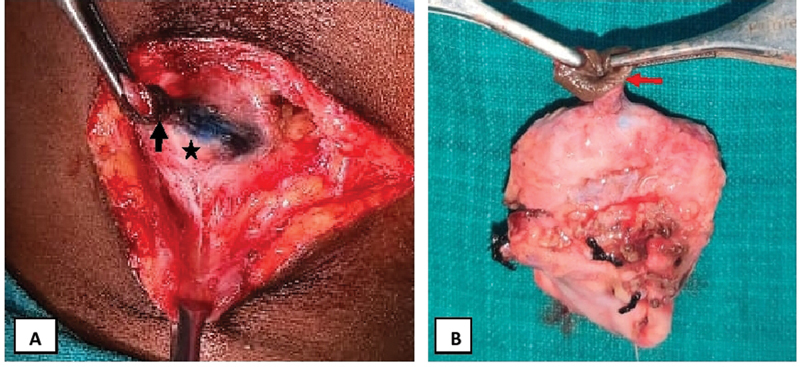
Intraoperative findings. (A) Fistula tract (black arrow) noted communicating with the underlying submandibular gland (black star). (B) Excised fistula (red arrow) with the rim of the surrounding skin on one end and submandibular gland on the other end.

**Fig. 3:**
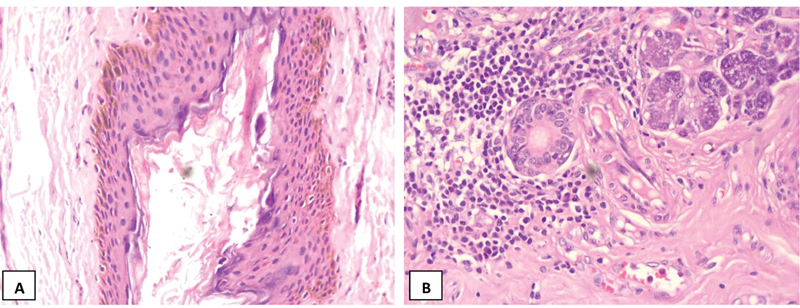
Histopathological findings. (A) Section showing fistula tract lined with squamous epithelium. (B) Section showing mild sialadenitis with lymphohistiocytic infiltrates.

An 18-year-old female presented with intermittent watery-to-purulent discharge from a small opening over the right submandibular neck region for 6 years. She had history of a small nodule over the same site, which had been previously manipulated by a local doctor. Since then, she noticed intermittent discharge from the opening that became profuse while eating or when she was exposed to the smell of food. It was associated with pain over the right submandibular neck region. On examination, a small opening was noted just lateral to the greater horn of the hyoid bone. Expression of serous discharge was noted on gentle palpation of the surrounding neck region. No signs of lymph nodal disease or stigmata of granulomatous disease were noted. Based on these findings, a clinical diagnosis of salivary fistula was suspected and a computed tomography (CT) with fistulography was performed after instilling the dye from the cutaneous opening. Computed tomography fistulography showed a vertical tract communicating with the right submandibular gland parenchyma. Multiple intraglandular dilated branching tracts (corresponding to salivary gland ductules) were also noted converging into a horizontal tract (Wharton duct) that communicated with the floor of the mouth ( Fig. 1A and 1B ). Diluted methylene blue dye was instilled through the cervical opening and was found extruding intraorally from the right Wharton duct ostia. These findings confirmed a diagnosis of right submandibular gland cutaneous fistula. The entire fistula tract with surrounding neck skin was excised along with the right submandibular salivary gland under general anesthesia ( Fig. 2A and 2B ). Histopathological examination of the excised surgical tissue showed a fistula tract lined by squamous epithelium. The deeper aspects of the tract showed a sparse chronic inflammatory infiltrate of lymphocytes and histiocytes in the surrounding stroma. The submandibular gland showed a sparse aggregate of lymphocytes in several lobules and around mildly dilated ducts ( Fig. 3A and 3B ).

## Methods

**Fig. 4:**
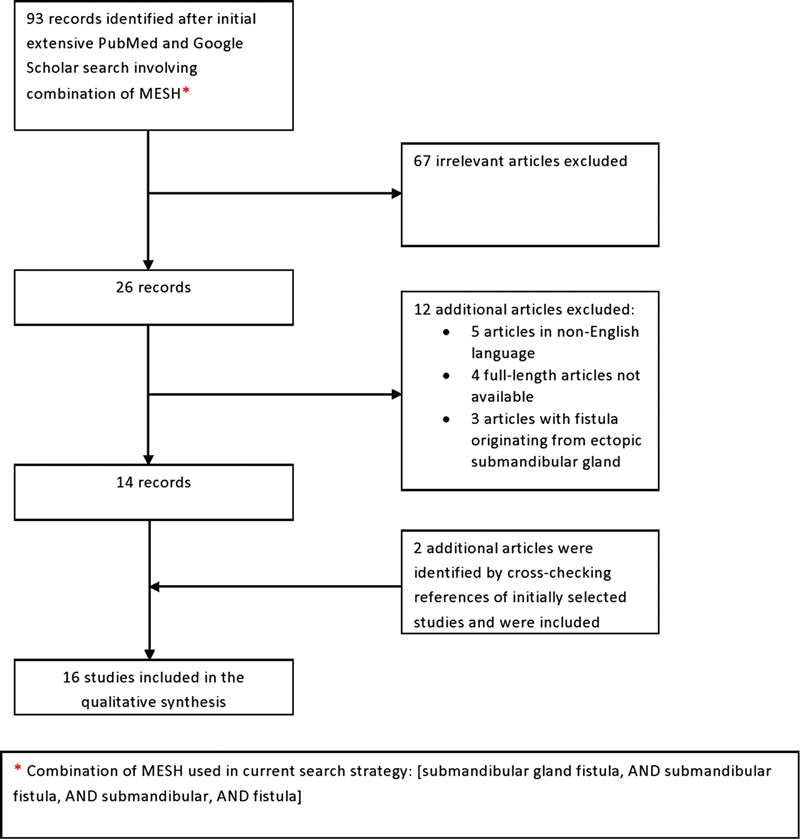
Literature search flow diagram based on the Preferred Reporting Items for Systematic Reviews and Meta-Analyses (PRISMA) standards.

A comprehensive review of the literature was performed using PubMed and Google Scholar database in September 2022 and reported in accordance with the Preferred Reporting Items for Systematic Reviews and Meta-Analyses (PRISMA) standards ( Fig. 4 ). The database was searched for full-length articles using a combination of keywords, *submandibular gland fistula* , AND *submandibular fistula* , AND *submandibular* AND *fistula* and compatible with submandibular gland/duct fistula. The content of each article was reviewed in order to identify the studies relevant to the topic. Cases with fistulae arising from aberrant/ectopic glands were excluded. Only articles published in English literature and confined to humans were included. No age limits were applied. Information from the included articles and an illustrative case were collected in a predesigned Microsoft excel spreadsheet (Microsoft Corp., Redmond, WA, USA). Continuous variables were summarized with mean and standard deviation (SD). Nominal variables were summarized with frequency and percentage. No other statistical tests were done.

## Result

A total of 18 cases (17 cases from 16 published articles and an illustrative case) were included in the current review ( Tables 1 and 2 ). 3 4 5 6 7 8 9 10 11 12 13 14 15 16 17 18 The age ranged from 6 to 72 years. The exact numerical age was not mentioned in two cases reported by Keiliszak et al. Of the remaining 16 cases, the average age was 39.75 (SD: 20.93) years. There were 12 males and 6 females (male/female ratio: 2/1). Calculi/foreign body (50%) and trauma (22.2%) were the 2 most common associated etiologies. Most fistulae had opening at the cervical cutaneous site (61.1%) followed by the mucosa of the floor of the mouth (16.7%).

**Table 1:** Summary of cases of submandibular gland fistula

Author ^Ref. #^	Year	Age (years)	Sex	Cause	Opening	Treatment
Geus et al. 3	1976	8	Male	Trauma (electric current)	Neck skin	Gland and fistula tract excision
Knezević et al. 4	1983	14	Male	Foreign body	Neck skin	Excision of external sinus & removal of glass
McFerran et al. 5	1993	41	Female	Congenital	Oropharynx	Observation- spontaneous regression
Paul et al. 6	1995	45	Male	Calculi	Neck skin and floor of mouth	Removal of sialolith and sialo-cutaneous fistula tract
Singh et al. 7	1995	41	Male	Trauma (gunshot)	Neck skin	Spontaneous resolution with conservative management
Obaid et al. 8	2000	49	Male	Calculi	Floor of mouth	Conservative management
Durgun et al. 9	2003	60	Female	Congenital	Neck skin	Gland and fistula tract excision
Jana et al. 10	2006	10	Male	Trauma	Neck skin	Gland and fistula tract excision
Saha et al. 11	2012	54	Male	Calculi	Neck skin	Gland and fistula tract excision, calculi excised
Saluja et al. 12	2012	65	Male	Calculi	Floor of mouth	Calculi excision and suture repair of fistula opening
Rangappa et al. 13	2014	55	Female	Calculi	Neck skin	Gland and fistula tract excision, calculi excised
Keiliszak et al. 14	2015	20s	Male	NK	Submucosal floor of mouth	Gland excision
Keiliszak et al. 14	2015	20s	Male	Calculi	Floor of mouth	Gland and fistula tract excision, calculi excised
Kulkarni et al. 15	2015	45	Male	Malignant tumor	Neck skin	Radical submandibular gland excision, segmental mandibulectomy and modified radical neck dissection.
Kusunoki et al. 16	2017	72	Male	Calculi	Neck skin	Gland and fistula tract excision, calculi excised
Stegmann et al. 17	2018	6	Female	Calculi	Neck skin	Sialendoscopic removal of sialolith and sialo-cutaneous fistula tract excision
Ha et al. 18	2019	53	Female	Sialadenitis	Subplatysmal neck site	Conservative management with systemic antibiotic
Present case	2022	18	Female	Trauma	Neck skin	Gland and fistula tract excision

**Table 2:** Etiology, clinical findings, radiological features, and advocated treatment amongst the reported cases of submandibular gland fistula

Variable	Number (n)	Frequency (%)
Cause •Calculi/ foreign body •Trauma (gun shot, electric current, physical, iatrogenic) •Congenital •Malignant tumor •Sialadenitis •NIO/NK	9 4 2 1 1 1	50 22.22 11.11 5.56 5.56 5.56
Fistula opening site •Isolated cutaneous neck •Isolated floor of mouth •Both cutaneous neck & floor of mouth •Oropharynx •Submucosal floor of mouth •Subplatysmal neck site	11 3 1 1 1 1	61.11 16.67 5.56 5.56 5.56 5.56
Clinical features •Discharge from fistula opening •Swelling / mass in submandibular region •Tender/pain over submandibular region •Throat lump	12 10 8 1	66.67 55.56 44.44 5.56
Imaging modality advocated •CT scan/ CT fistulography •USG •Xray •Xray sialography/ fistulography •OPG •Barium swallow •NP	6 2 3 4 1 1 3	33.33 11.11 16.67 22.22 5.56 5.56 16.67
Radiological findings •Fistula tract •Calculi	6 6	40 (6/15)* 66.67 (6/9) □
Treatment modality Surgical •Gland and fistula tract excision with/without calculi removal •Gland preserving surgery (calculi/foreign body removal with fistula excision/repair) Conservative management/ observation	14 10 4 4	77.78 55.56 22.22 22.22

Abbreviations: NIO/NK, no information obtained/ not known; NP, not performed.

*Total number of cases amenable to radiological investigations in which fistula were detected.

□ Total number of calculous causes of fistula in which calculi were detected radiologically.

Clinical symptoms/signs were present in the following frequencies: Discharge from fistula opening (66.7%), swelling/mass in the submandibular region (55.6%), tender/pain over the submandibular region (44.4%), and throat lump (5.6%). Three out of 12 discharging fistula cases (25%) had discharge symptoms that got aggravated while eating or when the patient was exposed to the smell/thought of food (Jana et al., Rangappa et al., and the present case). In the remaining nine cases, no relationship between discharge and food was mentioned or present. One case presented a sensation of throat lump that increased with stress (McFerran et al.).

Clinical manifestation varied with the site of fistula opening and associated etiology. External fistulae mostly presented with serous/mucoid/mucopurulent discharge from the opening, which can sometimes be related to food. Eleven out of a total of 12 patients (91.67%) with external cutaneous opening complained of discharging fistulae. Only one case of external cutaneous fistula did not mention symptom of discharge (Kusunoki et al.). Most patients with calculi/foreign body as etiology presented with obstructive complaints of pain/tenderness or swelling in the submandibular region. Out of 9 cases with sialolith/ductal foreign body, 7 (77.78%) presented with either both or isolated symptom of pain/tenderness and/or swelling over the submandibular site. The remaining two cases of calculi/foreign body only presented with discharge (Knezević et al. and Saha et al.).

Fifteen cases advocated various imaging modalities including plain radiogram, X-ray sialography/fistulography, orthopantomogram (OPG), barium swallow, ultrasonography (USG) and CT scan/CT fistulography. In the remaining three cases, no radiological investigation was done. In these 15 cases, a radiological diagnosis of fistula was made in only 6 patients (40%). Amongst the 9 cases with actual calculi, a radiological detection was made in 6 cases (66.7%).

Gland excision with removal of fistula and/or calculi was the most commonly advocated treatment (55.6%). Gland preserving surgery and conservative management were less commonly performed and both were utilized in 22.2% of cases.

## Discussion

Submandibular gland fistula is a rare entity. It is associated with various acquired etiologies, including calculi, foreign body, trauma, neoplasm, sialadenitis, or a congenital process linked to the branchial apparatus. 3 4 5 6 7 8 9 10 11 12 13 14 15 16 17 18

It can be an external sialo-cutaneous fistula or an internal sialo-oral fistula. The external opening is either cutaneous or subcutaneous. Similarly, an internal fistula opening is either mucosal or submucosal. 3 4 5 6 7 8 9 10 11 12 13 14 15 16 17 18

Clinical manifestation varies with the site of fistula opening and associated etiology. An external fistula mostly presents with serous/mucoid/mucopurulent discharge from the opening, which can sometimes be related to food. On the other hand, a fistula associated with calculi/foreign body mostly presents with obstructive or colicky complaints of pain/tenderness or swelling in the submandibular region.

Various authors have advocated different forms of management varying from observation and conservative management to surgical intervention, including either gland-preserving surgery or excision of the gland with fistulae tract. 3 4 5 6 7 8 9 10 11 12 13 14 15 16 17 18

## Final Comments

- The present systematic review highlights certain vital clinical and radiological clues that will aid in an early diagnosis of submandibular gland fistula even prior to surgical exploration.
- We believe that these diagnostic checkpoints will help clinicians achieve optimal management planning and avoid irrelevant investigations/interventions, especially in a resource-limited hospital setting.
